# Two Novel Biallelic Variants in the *FARSA* Gene: The First Italian Case and a Literature Review

**DOI:** 10.3390/genes15121573

**Published:** 2024-12-05

**Authors:** Sonia Lomuscio, Dario Cocciadiferro, Francesco Petrizzelli, Niccolò Liorni, Tommaso Mazza, Annalisa Allegorico, Nicola Ullmann, Giuseppe Novelli, Renato Cutrera, Antonio Novelli

**Affiliations:** 1 Department of Biomedicine and Prevention, University of Rome Tor Vergata, 00133 Rome, Italy; novelli@med.uniroma2.it; 2 Medical Genetics Unit, Tor Vergata University Hospital, 00133 Rome, Italy; 3 Translational Cytogenomics Research Unit, Bambino Gesù Children’s Hospital, IRCCS, 00165 Rome, Italy; dario.cocciadiferro@opbg.net (D.C.); t.mazza@operapadrepio.it (T.M.); antonio.novelli@opbg.net (A.N.); 4 Laboratory of Bioinformatics, IRCCS Casa Sollievo della Sofferenza, 71013 S. Giovanni Rotondo, Italy; f.petrizzelli@operapadrepio.it (F.P.); n.liorni@operapadrepio.it (N.L.); 5 Department of Experimental Medicine, Sapienza University of Rome, 00161 Rome, Italy; 6 Unit of Pediatric Pneumology and UTSIR, Santobono-Pausillipon Hospital, 80129 Naples, Italy; aallegorico@gmail.com; 7 Paediatric Pulmonology and Cystic Fibrosis Unit, Bambino Gesù Children’s Hospital, IRCCS, 00165 Rome, Italy; nicola.ullmann@opbg.net (N.U.); renato.cutrera@opbg.net (R.C.)

**Keywords:** *FARSA*, nintedanib, interstitial lung disease

## Abstract

**Background/Objectives**: The *FARSA* gene encodes for the catalytic α subunit of Cytoplasmic phenylalanine-tRNA synthetase (FARS1), an essential enzyme for protein biosynthesis in transferring its amino acid component to tRNAs. Biallelic pathogenic variants have been associated with a multisystemic condition, characterized by variable expressivity and incomplete penetrance. Here, we report the case of an 11 year-old girl presenting interstitial lung disease, supratentorial leukoencephalopathy with brain cysts, hepatic dysfunction, hypoalbuminemia, skin and joint hyperlaxity, growth retardation, and dysmorphic features. In addition, our patient also developed two clinical features never reported before: hypergammaglobulinemia and myopic chorioretinitis. **Methods**: NGS analysis of the patient’s skin-derived DNA revealed two novel biallelic variants in *FARSA* gene (NM_004461.3) never described before: the maternal nonsense variant, c.799C>T [p.(Gln267Ter)], and the paternal missense variant, c.737T>C [p.(Met246Thr)], both predicted as deleterious. **Results**: From a therapeutic perspective, this young girl has been enrolled in a clinical trial with Nintedanib, in order to treat the severe pulmonary fibrosis, with interesting initial results. **Conclusions**: Our findings expand the clinical and molecular spectrum of the *FARSA*-related phenotype and introduce new cues on lung fibrosis treatment in pediatric age.

## 1. Introduction

Aminoacyl-tRNA synthetases (ARSs) play an important role in cellular translational processes [1], providing the covalent ligation of tRNAs with their specific amino acids [2]. According to their localization, it is possible to categorize ARSs into two groups: cytoplasmic ARSs and mitochondrial ARSs. ARSs(Mito) mediate the synthesis of a dozen proteins involved in oxidative phosphorylation, whereas ARSs(Cyto) regulate the synthesis of a large part of intracellular proteins [3]. In detail, cytoplasmic ARS is a heterodimeric complex composed of an α-subunit with a catalytic function (FARSA) and a β-subunit with a regulatory role (FARSB), together forming a 4-helix bundle interface [4]. To date, almost 60 different ARSophaty have been identified, whether autosomal dominant or recessive forms [1]. Biallelic variants in *FARSB* were demonstrated to be causative of Rajab syndrome (MIM#613658), which presents with hypotonia, psychomotor retardation, liver dysfunction, and involvement of the lung and skeletal systems. A similar phenotype in a boy with biallelic variants in *FARSA* was first described in 2019 [5]. As a matter of fact, the detection of biallelic pathogenic variants in the *FARSA* gene matches a multisystemic condition (Rajab interstitial lung disease with brain calcifications-2, MIM #619013), characterized by variable expressivity and incomplete penetrance. To date, 11 cases of the abovementioned syndrome have been reported in the literature, with a wide variety of clinical manifestations [6,7].

Here, we describe the first Italian patient with Rajab interstitial lung disease with brain calcifications-2, presenting a multi-organ involvement in line with *FARSA*-related phenotype and, in addition, two clinical signs never mentioned before. Our findings expand the clinical and molecular spectrum of the *FARSA*-related phenotype, introducing new hints in pediatric treatment.

## 2. Materials and Methods

### 2.1. Genetic Studies

After obtaining informed consent for the genetic analyses, targeted exome enrichment and parallel sequencing were performed on genomic DNA extracted from the proband’s fibroblasts and circulating leukocytes of the unaffected parents.

### 2.2. Clinical Exome Sequencing

Library preparation and clinical exome capture were performed by using the ClinEX pro kit (4bases, Suglio, Switzerland) according to the manufacturer’s protocol and sequenced on the Illumina NovaSeq 6000 platform (Illumina, San Diego, CA, USA). The BaseSpace pipeline and Geneyx software v5.17 (LifeMap Sciences, Covina, CA, USA) were, respectively, used for variant calling and annotation. Sequencing data were aligned to the hg19 human reference genome. The global minor allele frequency (MAF) for the analyzed variants was obtained from the Genome Aggregation Database (gnomAD ver. 2.1.1). Based on the guidelines of the American College of Medical Genetics and Genomics (ACMG), a minimum depth coverage of 30× was considered suitable for analysis. Variants were visualized using the Integrative Genomics Viewer software (IGV) v2.18.4.

### 2.3. Effect and Prediction

p.[Met246Thr] was first evaluated in silico through an array of 11 pathogenicity predictors and 2 conservation scores. In particular, their potential pathogenicity was evaluated using AlphaMissense, CADD v1.7, DANN v1.0.0, DEOGEN 2, FATHMM-MKL coding v2.3, LRT (released on November 2009), M-CAP v1.3, MutationTaster v2, MetaLR (accessed in September 2024), SIFT4G v2.4, and VEST v4.0. Conservation was assessed using GERP and PhyloP (accessed in September 2024). p.[Gln267Ter] was evaluated by its effect on Nonsense Mediated Decay by using the masonmd v.1.10.0 tool [8].

Atomic coordinates of the human *FARSA* protein in complex with *FARSB* were retrieved from the RCSB Protein Data Bank (PBD_ID: 3l4g). Then, structural damages caused by p.[Met246Thr] were characterized using the Missense3D web tool (accessed in September 2024) [9]. The stability of the mutant FARSA-FARSB complex was investigated thermodynamically through the BuildModel function implemented in FoldX v5.0 [10,11], run with standard parameters.

## 3. Results

### 3.1. Clinical Description

**Figure 1:**
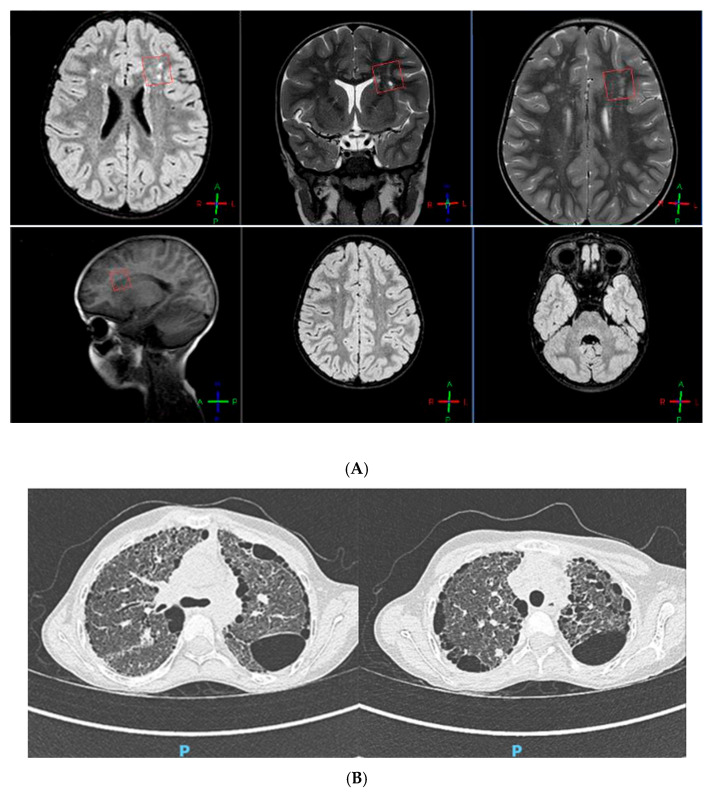
(**A**) Brain MRI showing leukoencephalopathy. (**B**) Thoracic CT scan showing “ground glass” and “honeycomb” patterns.

Our patient was an 11-year-old girl, the only child of non-consanguineous, healthy parents. She was born at term (40 weeks of gestation), with a weight of 2.880 kg (15th centile). No anomalies were observed at the transfontanellar ultrasound screening. Her mother referred lactation difficulties (weak sucking reflex) due to muscular hypotonia. Consequently, the girl developed failure to thrive. At 1-month-old age, she was hospitalized for a urinary tract infection. She also started to suffer of frequent upper airway infections, associated with bronchospasm. In the first years of life, a delay in reaching motor development milestones was detected (walking was achieved at 21 months). Hence, by the age of 2, the girl underwent her first brain magnetic resonance imaging (MRI), which showed signs of leukomalacia; then, a neuropsychiatric evaluation and an EEG were performed, with normal results. Given the co-presence of poor growth and psychomotor retardation, chromosome analysis was carried out: karyotype was normal (46, XX), while the Chromosomal Microarray Analysis (CMA) identified a paternally inherited microduplication of the 19q13.41 region, with no pathogenic significance. At the age of 6, a brain MRI was repeated, showing “white matter lesions in the fronto-temporo-parietal regions, encysted perivascular spaces and mega cisterna magna” (Figure 1A).

An echocardiographic assessment revealed “the presence of ascending aortic ectasia and a systemic-to-pulmonary small collateral vessel, causing a left-to-right shunt with no hemodynamical relevance”. At 7 years old, the girl had a moderate restrictive ventilatory failure (FEV1 52%, FVC 51%, FEV1/FVC 101%). The 6 min walk test (6MWT) showed a decreased functional capacity with exercise-induced oxygen desaturation. Laboratory tests stressed a chronic condition of leukocytosis, hypoalbuminemia (microcytic anemia), and hypergammaglobulinemia; in addition, a persistent alteration of the hepatic enzymes values was reported. No hearing impairment was detected at the audiometry. Myopic chorioretinitis was observed at the fundoscopy. The orthopedic examination stressed the presence of bilateral flatfoot, scoliosis, and knee osteopenia. Based on the medical history marked by frequent respiratory infections and chronic cough, at the age of 10, the patient had a thoracic computed tomography (CT) scan, describing “lung fibrosis (ground glass pattern) with several subpleural cysts (honeycomb pattern), heterogeneously distributed”. As confirmation of these findings, spirometry was re-performed, highlighting a severe restrictive pulmonary insufficiency (FEV1 24%, FVC 27%).

In order to prevent a further worsening of the pulmonary fibrosis, the young girl was enrolled in a clinical trial with Nintedanib, a tyrosine kinase inhibitor targeting endothelial growth factor receptor (VEGFR), platelet-derived growth factor receptor (PDGFR), and fibroblast growth factor receptor (FGFR) [12].

After 18 months of treatment with Nintedanib (25 mg B.I.D.), her respiratory function remained stable (FEV1 26%, FVC 28%). Likewise, a thoracic CT scan was repeated, pointing out a very slight enlargement of the already-known bronchiectasis and cysts (Figure 1B).

In parallel, genetic tests to rule out immuno-hematological disorders and surfactant deficiency were carried out and, in fact, their results were negative.

For a more accurate diagnostic definition, a skin biopsy was opted for. At the electron microscopy examination, a connective tissue alteration merged due to the detection of several elastic fibers with a small diameter (0.5–1.5 µm) located in proximity of fibroblasts. On elastic fibers surface, which appeared irregular with deep indentations, there were numerous disorganized microfibrils.

**Figure 2:**
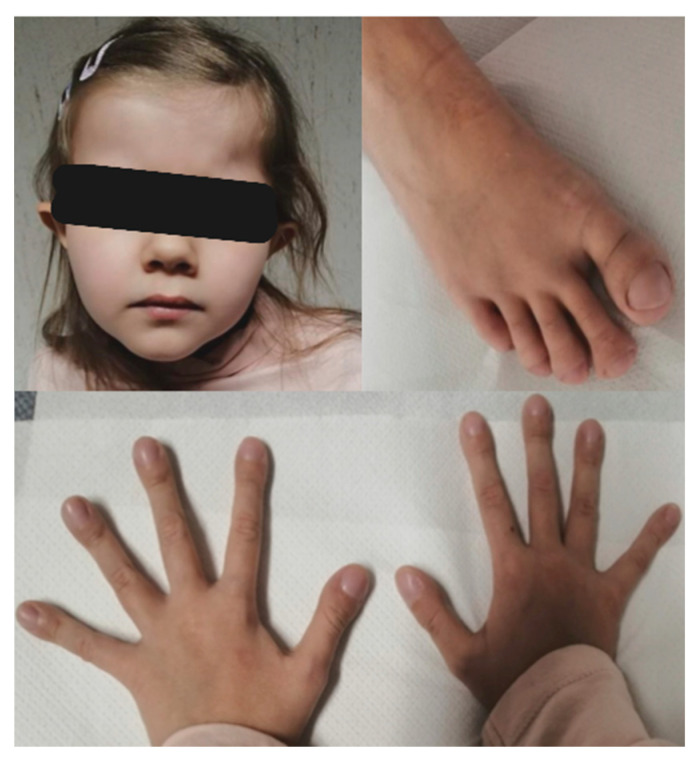
Facial dysmorphisms and digital clubbing.

Recent auxological parameters confirm the patient’s poor growth status: weight 16 kg (below 3rd centile, −3.79 SD), height 114.8 cm (below 3rd centile, −4.07 SD), CC 49.7 cm (below 3rd centile, −2.37 SD). We also report her dysmorphic features: elfin-like face, prominent forehead with frontal bossing, deeply set eyes, protruding ears with attached lobes, pectus carinatum, abnormal subcutaneous fat deposition at hips, arachnodactyly with digital clubbing (Figure 2).

### 3.2. Genetic and Bioinformatic Analyses

Trio-targeted exome sequencing data analysis revealed two compound heterozygous variants in the *FARSA* gene (NM_004461.3)—c.799C>T; p.(Gln267Ter) and c.737T>C; p.(Met246Thr)—inherited from the mother and the father, respectively. The nonsense variant p.Gln267Ter causes a premature termination codon in position c.799 and the consequent length of the mutated coding sequence is 801bp versus 1524bp of *FARSA* wildtype. It has never been reported. It is conserved through Vertebrates and Mammalia, and is predicted not to block the NMD (NMD-elicited mutation) in silico. Moreover, it can be classified as likely pathogenic according to the American College of Medical Genetics and Genomics (ACMG) criteria PM2 and PVS1 (% protein length lost = [(508 − 267Ter)/508total] × 100% = 47.44% loss) (Supporting Information) [13,14].

The missense variant p.Met246Thr has never been reported. It is phylogenetically conserved and was predicted to be disease-causing by 10 software predictors out of 11 (Supporting Information), showing high congruency in the overall assessment [15]. Coherently, it can also be classified as likely pathogenic based on the ACMG criteria including PM1, PM2, PP2, and PP3. Finally, the p.Met246Thr substitution seems to remarkably energetically destabilize the FARSA-FARSB complex, with a resulting ΔΔG (Met246Thr) = ΔGmut − ΔGwt = 2.41212 kcal/mol (Supporting Information).

## 4. Discussion

Here, we describe an Italian patient affected by interstitial lung disease, supratentorial leukoencephalopathy with brain cysts, hepatic dysfunction, hypoalbuminemia, skin and joint hyperlaxity, growth retardation, and dysmorphic features harboring the never-described before variants in compound heterozygous state in the *FARSA* gene: (NM_004461.3): c.799C>T; p.(Gln267Ter) and c.737T>C; p.(Met246Thr). To date, 13 *FARSA* missense variants have been associated with Rajab interstitial lung disease with brain calcifications-2, although the relationship between the phenotype and gene is still provisional.

This is the first Italian/twelfth worldwide patient with biallelic mutations in the *FARSA* gene, and the first harboring a truncating variant. The nonsense p.(Gln267Ter) variant is predicted in silico not to block the NMD process (NMD-elicited mutation), while the p.Met246Thr variant resulted in significantly impacting the FARSA-FARSB complex stability.

It is possible to state that all the published patients presented an interstitial lung disease, complicated by cholesterol pneumonitis in eight cases out of twelve. In half of the cases, pulmonary cysts were detected (6/12). Due to worsening of the lung respiratory function, digital clubbing was present in eight cases out of twelve. Poor growth was referred to in 10/12, often associated with gastrointestinal disturbances (diarrhea and/or vomiting). Also, the liver is affected in 7/12 cases by steatosis and/or fibrosis, with a consequential alteration of hepatic enzymatic values. In four patients, chronic cytolysis and cholestasis were reported too. After the respiratory system, the second most involved is the nervous one: nine children developed speech and/or motor delay, seven had their head circumference below 3rd centile, four had white matter lesions, four cerebral vasculopathy, and three presented brain cysts. At birth and in the first years of life, eight out of twelve children were hypotonic. Among clinical manifestations, an endocrine off-balance could be expected: 4 patients developed hypothyroidism and 3/12 had a Growth Hormone (GH) disequilibrium. In five cases, structural cardiovascular defects were highlighted.

Certainly, a clinical hallmark is the detection of hypoalbuminemia (11/12), followed by the alterations of the blood cell count (9/12).

A peculiar facial and body appearance is described: half of the patients had pectus carinatum and arachnodactyly, with a pronounced joint hyperflexibility (in a few cases related to cutis laxa). In 7 patients, an abnormal distribution of the subcutaneous fat tissue was described.

Among other less frequent manifestations, we point out osteopenia (2/12), sensorineural hearing loss (1/12), and kidney involvement (e.g., nephrolithiasis 2/12). As a non-specific eye involvement and chronic inflammation were previously reported, we point out two novel clinical features that showed up in our case: myopic chorioretinitis and hypergammaglobulinemia (Table 1).

In light of what emerged, resorting to Nintedanib was a targeted therapeutic choice. It is a tyrosine kinase inhibitor binding multiple signaling receptors, such as endothelial growth factor receptor (VEGFR), platelet-derived growth factor receptor (PDGFR), and fibroblast growth factor receptor (FGFR); it is therefore capable of inhibiting fibroblast proliferation and differentiation [12]. Currently, our hospital is conducting a clinical trial on Nintedanib as a primary treatment of idiopathic interstitiopathies in pediatric age. Due to her gradual lung function deterioration, our proband was enrolled in this pilot study, with early promising results about slowing the progression of lung fibrotization, as emerged from morphofunctional lung assessment (CT scan and spirometry) after only 18 months of therapy. The perception of clinical stability was corroborated by a Fan severity score of 3 (the patient was symptomatic with normal resting room air saturation, but abnormal saturation (SaO2 < 90%) with sleep or exercise) and by an average value of 2 on PedsQL questionnaires, administered to the girl and her parents, during the treatment monitoring phase [16].

**Table 1:** FARSA-related clinical manifestations and literature review.

		Index Patient (Presented Here)	1 patient Described [6]	4 Patients Described [17]	1 Patient Described [18]	3 Patients Described [1]	1 Patient Described [5]	1 Patient Described [7]	Σ
Variants		c.737T>C, c.799C>T	c.1172 T>C, c.1211G>A	P1: c.883C>T, c.883C>T; P2:c.883C>T, c.883C>T; P3:c.1066G>A, c.1066G>A; P4:c.1066G>A, c.1066G>A	c.1040C>T, c.1424G>A	P1: c.1210C>T, c.1254G>C; P2: c.883C>T, c.883C>T; P3: c.829T>G, c.829T>G	c.766T>C, c.1230C>A	c.982A>G, c.1109T>C	
Respiratory system	interstitial lung disease	yes	yes	4	yes	3	yes	yes	12/12
	cholesterol pneumonitis/foamy macrophages			4		3	yes		8/12
	pulmonary alveolar proteinosis					1			1/12
	cystic lung disease	yes			yes	2	yes	yes	6/12
	digital clubbing	yes		4		2	yes		8/12
Growth and Nutrition	failure to thrive	yes	yes	4	yes		yes	yes	9/12
	feeding difficulty/diarrhea/vomiting	yes	yes	2	yes	3	yes	yes	10/12
Liver	hepatomegaly/hepatosplenomegaly		yes	3	yes	2	yes		8/12
	Chronic cytolysis and cholestasis			4					4/12
	abnormal liver values (blood)	yes	yes		yes	3	yes	yes	8/12
	liver steatosis/hyperechogenicity		yes	2	yes	2	yes		7/12
Nervous system	hypotonia	yes	yes	1	yes	2	yes	yes	8/12
	neurodevelopmental (speech delay/ motor delay)	yes	yes	3	yes	2	yes		9/12
	microcephaly	yes	yes	4				yes	7/12
	brain cysts	yes				1	yes		3/12
	brain calcifications				yes		yes		2/12
	Cerebral vasculopathy			3		1			4/12
	stroke			2					2/12
	white matter lesions	yes	yes		yes	1			4/12
	brain aneurysm			2					2/12
Skeletal system	pectus carinatum/excavatum	yes		2		1	yes		5/12
	joint hyperflexibility	yes		2		2	yes	yes	6/12
	Scoliosis	yes					yes		2/12
	Aseptic osteomyelitis			1					1/12
	osteopenia	yes				1			2/12
	Arachnodactyly	yes		2		1	yes		5/12
Endocrine system	growth hormone resistance/deficiency			1		2			3/12
	Adrenal insufficiency				yes				1/12
	Hypopituitarism						yes		1/12
	Micropenis, cryptorchidy			1					1/12
	hypothyroidism	yes	yes		yes		yes		4/12
Dysmorphic features	face/body appearance	yes	yes	4		1	yes		8/12
Eye	abnormal eye movement/nystagmus					1			1/12
	reticular opacities					1			1/12
	myopic chorioretinitis	yes							1/12
Ear	sensorineural hearing loss					1			1/12
Cardiovascular system	structural heart/vessel defects	yes	yes			2		yes	5/12
Immune system	abnormal blood cell counts/leucocytosis	yes	yes	4	yes		yes	yes	9/12
	hypergammaglobulinemia	yes							1/12
	hypoalbuminemia	yes	yes	4		3	yes	yes	11/12
Urinary system	vesicoureteral reflux						yes		1/12
	nephrolithiasis					1		yes	2/12
	tubulopathy				yes	1			2/12
	proteinuria				yes				1/12
Skin/Connective Tissue	poor wound healing/frail skin			2		1			3/12
	Cutis laxa	yes		2					3/12
	hernia (Abdominal/Inguinal)			2			yes		3/12
	abnormal subcutaneous fat tissue	yes		4		1	yes		7/12

## 5. Conclusions

We have made a further contribution in outlining the *FARSA*-related phenotypical spectrum through the analysis of sign and symptom frequencies. It follows that interstitiopathy is a crucial implication for all affected individuals. A preliminary examination of Nintedanib effects on our patient suggests that a trend reversal can be induced in lung fibrosis exacerbation. Our study expands the clinical and molecular spectrum of the *FARSA*-related phenotype and introduces new cues on lung fibrosis treatment in pediatric age. However, due to the current limited number of known *FARSA* variants and the multi-trait nature of the disease, it is necessary to confirm these assessments as new variants emerge, in order to validate these findings and to understand the full spectrum of clinical manifestations associated with *FARSA* mutations. Although based on the medical history of one single patient, we hope that the information provided on the diagnostic and therapeutic path we have chosen may lead to the rapid identification and treatment of other affected subjects. In this way, as more *FARSA*-related cases are reported in the literature, it will be possible to better manage the disease and to accurately predict its prognosis.

## Data Availability

The original contributions presented in this study are included in the article/Supplementary Materials. Further inquiries can be directed to the corresponding author.

## Supplementary Materials

The following supporting information can be downloaded at https://www.mdpi.com/article/10.3390/genes15121573/s1, Table S1: In-silico pathogenicity and phylogenetic conservation predictions for p.[Met246Thr]. Four software packages did not return an estimate. Figure S1: Atomic coordinates of the human FARSA protein (in cyan) in complex with FARSB (in yellow). The p.(Met246Thr) mutant site is highlighted in red.
