# Supplementary material for: Two Novel Biallelic Variants in the FARSA Gene: The First Italian Case and a Literature Review
Source: Genes (Basel). 2024 Dec 5;15(12):1573. doi: 10.3390/genes15121573 (PMC11675362; doi:10.3390/genes15121573)
Supplement: Supplementary file 1 [file genes-15-01573-s001.zip › genes-3347335-supplementary.pdf]

Effect and prediction

p.[Met246Thr] was first evaluated in silico through an array of 11 pathogenicity predictors and 2 conservation scores. In particular, their potential pathogenicity was evaluated using AlphaMissense, CADD phred v1.7, DANN, DEOGEN 2, FATHMM-MKL coding, LRT (released on November 2009), M-CAP v1.3, MutationTaster v2, MetaLR, SIFT4G v2.4, and VEST v4.0. Conservation was assessed using GERP and PhyloP. p.[Gln267Ter] was evaluated by its effect on Nonsense Mediated Decay by masonmd tool [doi:10.1038/ncomms15943].

**In-silico pathogenicity predictions**	
AlphaMissense	0.979 (likely pathogenic)
CADD 1.7 phred	24.2 (D)
DANN score	0.94 (D)
DEOGEN 2	T
FATHMM-MKL coding	D
LRT	D
M-CAP	D
MutationTaster	D
MutationAssessor	.
MetaLR	D
PROVEAN	.
Polyphen2 HDIV	.
SIFT	.
SIFT4G	D
VEST4	0.896 (D)
**Conservation**	

GERP++ RS	2.68 (constrained)
PhyloP	6.73 (highly conserved)

Table S1: *In-silico* pathogenicity and phylogenetic conservation predictions for p.[Met246Thr]. Four software packages did not return an estimate.

Atomic coordinates of the human FARSA protein in complex with FARSB were retrieved from the RCSB Protein Data Bank (PBD_ID: 3l4g). Then, structural damages caused by p.[Met246Thr] were characterized using the Missense3D web tool. The stability of the mutant FARSA-FARSB complex was investigated thermodynamically through the BuildModel function implemented in the FoldX algorithm run with standard parameters.

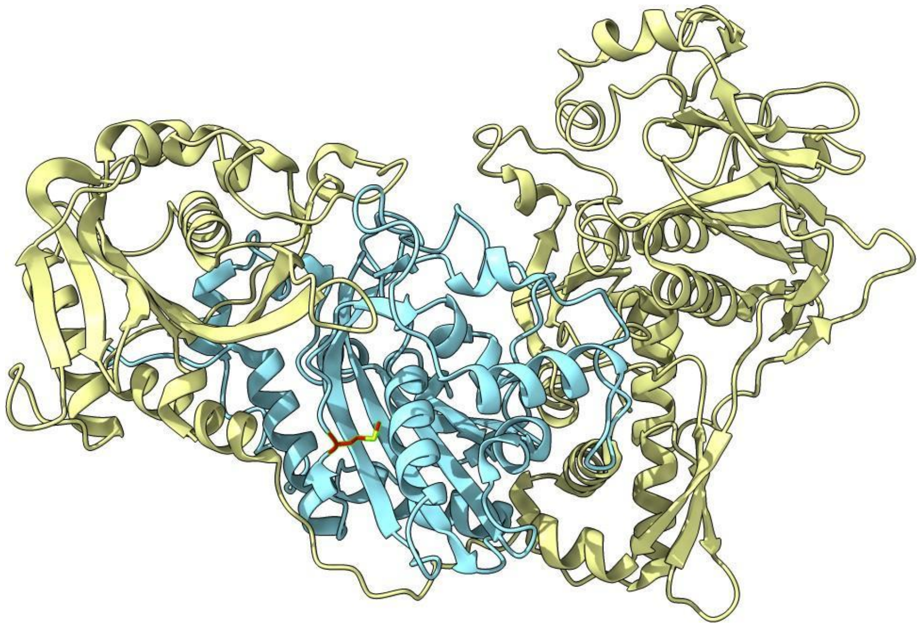

Figure S1: Atomic coordinates of the human FARSA protein (in cyan) in complex with FARSB (in yellow). The p.(Met246Thr) mutant site is highlighted in re
